# Differences and homologies of chromosomal alterations within and between breast cancer cell lines: a clustering analysis

**DOI:** 10.1186/1755-8166-7-8

**Published:** 2014-01-23

**Authors:** Milena Rondón-Lagos, Ludovica Verdun Di Cantogno, Caterina Marchiò, Nelson Rangel, Cesar Payan-Gomez, Patrizia Gugliotta, Cristina Botta, Gianni Bussolati, Sandra R Ramírez-Clavijo, Barbara Pasini, Anna Sapino

**Affiliations:** 1 Department of Medical Sciences, University of Turin, Via Santena 7, 10126 Turin, Italy; 2 Department of Laboratory Medicine, Azienda Ospedaliera Città della Salute e della Scienza di Torino, Turin, Italy; 3 Natural and Mathematical Sciences Faculty, Universidad del Rosario, Bogotá, Colombia; 4 Medical Genetics Center, Department of Cell Biology and Genetics, Center of Biomedical Genetics, P.O. Box 1738, 3000 DR Erasmus MC, Rotterdam, The Netherlands

**Keywords:** Cytogenetic, Chromosomal abnormalities, Breast cancer cell lines, Hierarchical cluster

## Abstract

**Background:**

The MCF7 (ER+/HER2-), T47D (ER+/HER2-), BT474 (ER+/HER2+) and SKBR3 (ER-/HER2+) breast cancer cell lines are widely used in breast cancer research as paradigms of the luminal and HER2 phenotypes. Although they have been subjected to cytogenetic analysis, their chromosomal abnormalities have not been carefully characterized, and their differential cytogenetic profiles have not yet been established. In addition, techniques such as comparative genomic hybridization (CGH), microarray-based CGH and multiplex ligation-dependent probe amplification (MLPA) have described specific regions of gains, losses and amplifications of these cell lines; however, these techniques cannot detect balanced chromosomal rearrangements (e.g., translocations or inversions) or low frequency mosaicism.

**Results:**

A range of 19 to 26 metaphases of the MCF7, T47D, BT474 and SKBR3 cell lines was studied using conventional (G-banding) and molecular cytogenetic techniques (multi-color fluorescence *in situ* hybridization, M-FISH). We detected previously unreported chromosomal changes and determined the content and frequency of chromosomal markers. MCF7 and T47D (ER+/HER2-) cells showed a less complex chromosomal make up, with more numerical than structural alterations, compared to BT474 and SKBR3 (HER2+) cells, which harbored the highest frequency of numerical and structural aberrations. Karyotype heterogeneity and clonality were determined by comparing all metaphases within and between the four cell lines by hierarchical clustering. The latter analysis identified five main clusters. One of these clusters was characterized by numerical chromosomal abnormalities common to all cell lines, and the other four clusters encompassed cell-specific chromosomal abnormalities. T47D and BT474 cells shared the most chromosomal abnormalities, some of which were shared with SKBR3 cells. MCF7 cells showed a chromosomal pattern that was markedly different from those of the other cell lines.

**Conclusions:**

Our study provides a comprehensive and specific characterization of complex chromosomal aberrations of MCF7, T47D, BT474 and SKBR3 cell lines.

The chromosomal pattern of ER+/HER2- cells is less complex than that of ER+/HER2+ and ER-/HER2+ cells. These chromosomal abnormalities could influence the biologic and pharmacologic response of cells. Finally, although gene expression profiling and aCGH studies have classified these four cell lines as luminal, our results suggest that they are heterogeneous at the cytogenetic level.

## Background

The MCF7, T47D, BT474 and SKBR3 breast cancer cell lines are commonly used in experimental studies of cellular function, and much of the current knowledge of molecular alterations in breast cancer has been obtained from these cell lines [1-4].

Whole-genome studies using microarray expression analyses have identified distinct subtypes of breast carcinomas (the luminal, HER2+, and basal-like subtypes) based on the expression of approximately 500 genes (the so-called “intrinsic gene list”) [5-7]. These molecular subtypes have been approximated using immunohistochemical markers. In this way, estrogen (ER) and progesterone receptor (PR)+/HER2- tumors are classified as belonging to the luminal A molecular subtype, ER+/PR+/HER2+ tumors to the luminal B subtype, ER-/PR-/HER2+ tumors to the HER2 subtype, and triple negative (ER-/PR-/HER2-) tumors to the basal-like carcinomas [8].

As determined by immunohistochemistry, the receptor profile classifies MCF7 and T47D cells (ER+/PR+/HER2-) as belonging to the luminal A subtype, BT474 cells (ER+/PR+/HER2+) as luminal B and SKBR3 cells (ER-/HER2+) as HER2 [9,10]. However, the RNA transcriptional profile determined by whole genome oligonucleotide microarrays [1,4,11] characterized all four-cell lines as luminal because of the expression of both ERα-regulated genes (e.g., MYB, RET, EGR3, and TFF1) [1] and genes associated with luminal epithelial differentiation (e.g., GATA3 and FOXA1).

Different works have assayed the DNA genetic profile of these cell lines using comparative genomic hybridization (CGH) and multiplex ligation-dependent probe amplification (MLPA) to describe many different copy number alterations [11-13]. With these techniques, however, balanced chromosome rearrangements (e.g., translocations or inversions) and low frequency mosaicism (< 30% abnormal cells) are not detectable. These chromosomal alterations may be assessed on metaphases using G-banding karyotype and multicolor fluorescence *in situ* hybridization (M-FISH) [2,12-16]. However, because both procedures are time consuming, they have been applied to only a small number of metaphases [2,12-17]. Thus, to our knowledge, a search for clonal chromosomal aberrations within each cell line [2,12-16] and a comprehensive comparison of the MCF7, T47D, BT474 and SKBR3 cell lines from a cytogenetic perspective have not yet been performed.

In the present study, we evaluated structural and numerical alterations on a large number of metaphases of MCF7, T47D, BT474 and SKBR3 breast cancer cell lines using a combination of G-banding and M-FISH. This allowed us to analyze cell clonality within each cell line and to thoroughly compare the cytogenetic of the cell lines by clustering analysis.

## Results

**Figure 1:**
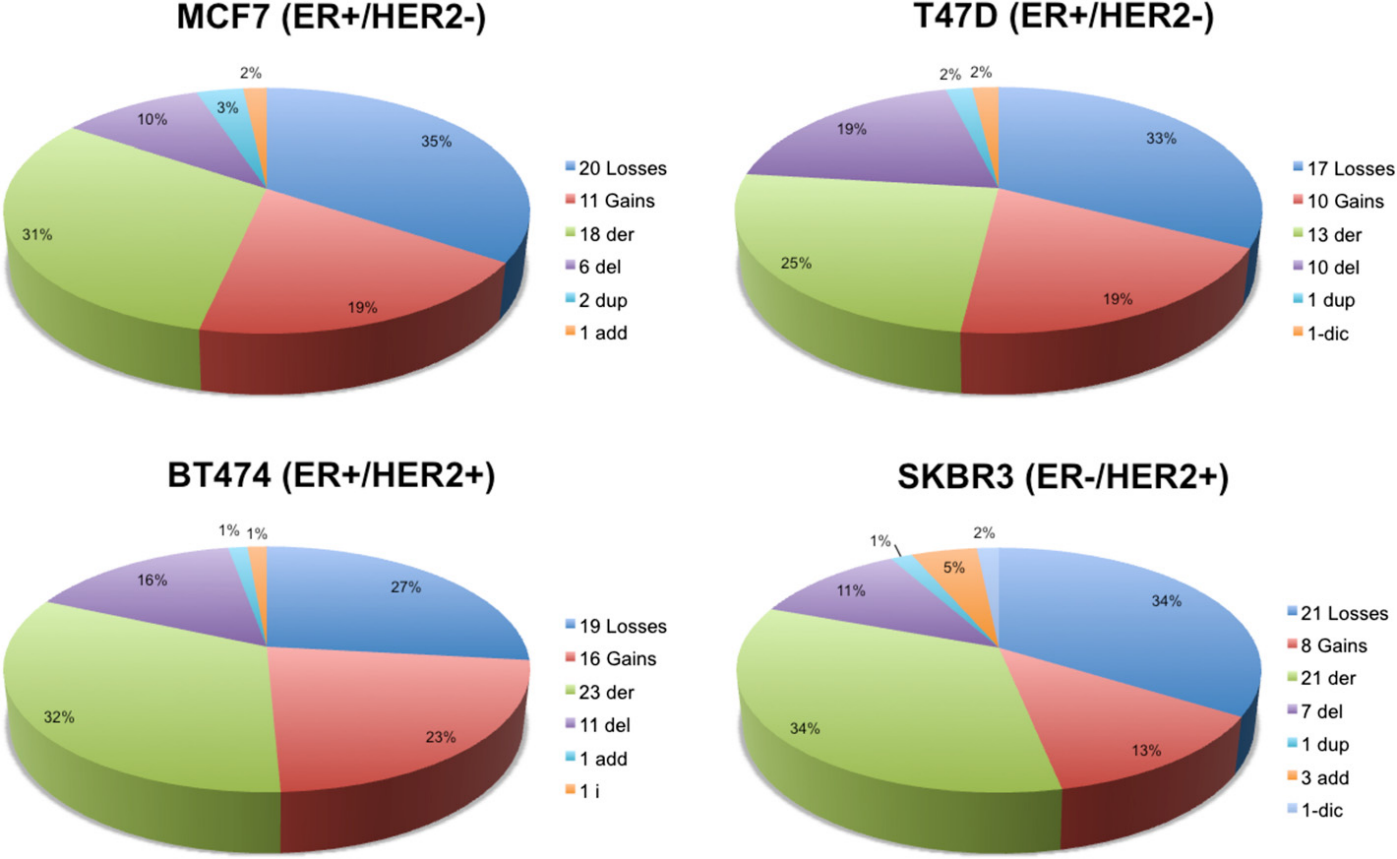
**Distribution of numerical and structural aberrations across the four breast cancer cell lines.** der = derivative chromosome; del = deletion; dup = duplication; add = additional material of unknown origin; dic = dicentric chromosome.

Between 19 and 26 metaphases with good chromosome dispersion and morphology were analyzed for each cell line to define the structural and numerical alterations, and 100 metaphases/cell line were analyzed to determine the level of ploidy. The rate and type of chromosomal abnormalities for each cell line are shown in Figure 1.

### Cytogenetic profile and cluster analysis of MCF7 cells

The cytogenetic analysis performed on 26 metaphases of MCF7 cells demonstrated a modal number hypertriploid to hypotetraploid (4n+/-) (76 to 88 chromosomes). Each chromosome harbored either a numerical or structural aberration, which accounted for 58 different rearrangements (31 numerical and 27 structural). Polyploidy was observed in 2% of the cells. Numerical alterations were present in all chromosomes; losses were more frequent than gains (Figure 1). Chromosomes 18 and 20 were nullisomic in 11.5% and 30.7% of the cells, respectively. Structural aberrations (translocations, duplications and deletions) were found in all chromosomes except 4, 5, 13, 14 and 18.

**Figure 2:**
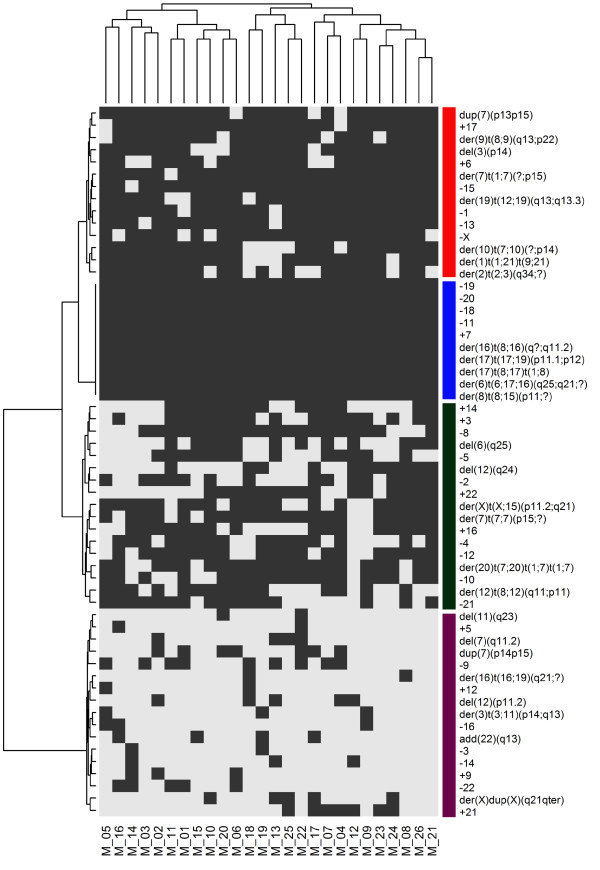
**Hierarchical cluster analysis of the presence or absence of chromosomal aberrations observed in 26 MCF7 metaphases.** Each column refers to a metaphase (M) and each row to a chromosomal abnormality. Grey indicates the presence of each abnormality, and white indicates their absence. The cluster number is indicated by vertical color bars. Cluster 1: red bar, cluster 2: blue bar, cluster 3: green bar and cluster 4: purple bar.

**Figure 3:**
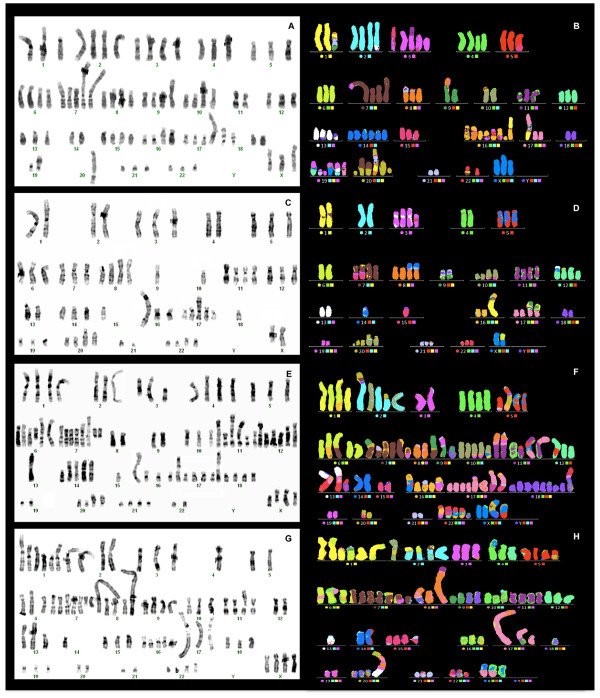
**G-Banding and molecular cytogenetic results of four breast cancer cell lines. A-B)** G-banded and M-FISH karyotype of a representative metaphase of MCF7 cells. **C-D)** G-banded and M-FISH karyotype of a representative metaphase of T47D cells. **E-F)** G-banded and M-FISH karyotype of a representative metaphase of BT474 cells. **G-H)** G-banded and M-FISH karyotype of a representative metaphase of SKBR3 cells.

A cluster analysis indicated that the types of chromosomal alterations were similar in the 26 metaphases (horizontal dendrogram, Figure 2). Clustering by the frequency of the chromosomal aberration within a cell line produced 4 clusters (vertical dendrogram, Figure 2). The first cluster (red bar) represented chromosomal alterations that were frequently present; chromosome 7 was the most affected by structural abnormalities. The second cluster (blue bar) represented alterations that were present in all metaphases, including chromosome losses and structural alterations of chromosomes 8 and 17. In particular, the loss of chromosomes 11, 18, 19 and 20 and the gain of chromosomes 7 and 17 were observed in all metaphases.der(6)t(6;17;16)(q25;q21;?), der(8)t(8;15)(p11;?), der(16)t(8;16)(q?;q11.2), der(17)t(8;17)t(1;8) and der(17)t(17;19)(p11.1;p12) were present in all cells as a consequence of structural aberrations (Table 1 and Figure 3A and 3B).

**Table 1:** G-Banding and M-FISH karyotypes of all breast cancer cell lines studied

**Cell line**	**Karyotype**
**MCF7**	76 ~ 88 < 4n>,-X[11],-Xx2[8],-Xx3[4],der(X)t(X;15)(p11.2;q21)[16],
	der(X)t(X;15)(p11.2;q21)x2[3],der(X)dup(X)(q21qter)[5],-1[22]-1x2[2], der(1)t(1;21)t(9;21)[22],-2[13],-2x2[2],der(2)t(2;3)(q34;?)[19],-3[2],
	+3[17],del(3)(p14)[22],der(3)t(3;11)(p14;q13)[3],-4[12],-4x2[4],
	+5[2],-5[13],+6[9],+6x2[8],+6x3[4],add(6)(q27)[2],del(6)(q25)[4],
	del(6)(q25)x2[8], der(6)t(6;17;16)(q25;q21;?)[26],
	+7[26],der(7)t(1;7)(?;p15)[23],der(7)t(1;7)(?;p15)x2[2], del(7)(q11.2)[4],dup(7)(p13p15)[7],dup(7)(p13p15)x2[5],dup(7)(p13p15)x3[11],dup(7)(p14p15)[5],dup(7)(p14p15)x2[2],der(7)t(7;7)(p15;?)[19], der(7)t(7;7)(p15;?)[2],-8[8], -8x2[12],der(8)t(8;15)(p11;?)[26],+9[3]
	-9[7],-9x2[2],der(9)t(8;9)(q13;p22)[22],-10[6],-10x2[10],-10x3[3],
	der(10)t(7;10)(?;p14)[9],der(10)t(7;10)(?;p14)x2[12],-11[14],
	-11x2[12],del(11)(q23)[2],-12[15],-12x2[4],+12[2],
	del(12)(p11.2)(5),del(12)(q24)[11],der(12)t(8,12)(q11;p11)[15],
	-13[12],-13x2[10],-13x3[2],-14[3],+14[14],-15[12],-15x2[10],
	-15x3[3],-16[3],+16[16],der(16)t(8;16)(q?;q11.2)[8],der(16)t(8;16)(q?;q11.2) x2[17]der(16)t(16;19)(q21;?)[2],+17[11],+17x2[10],+17x3[5],der(17)t(8;17)t(1;8)[21],der(17)t(8;17)t(1;8)x2[5],der(17)t(17;19)(p11.1;p12)x2[17],-18[4],
	-18x2[14],-18x3[5],-18x4[3],-19[7],-19x2[15],-19x3[4],
	der(19)t(12;19)(q13;p13.3)[21],der(19)t(12;19)(q13;p13.3)x2[2],-20[2],
	-20x2[5],-20x3[11],-20x4[8],der(20)t(7;20)t(1;7)t(1;7)[21],+21[5],+21x2[2],-21[14],-21x2[2],+22[12],+22x2[3],-22[3],-22x2[2],add(22)(q13)[4][cp26]
**T47D**	57 ~ 66 < 3n>,X,-X[24],der(X)t(X;6)(q12;p11)[24],-1[19],-2[22],
	-3[5],del(3)(p11)[2],del(3)(p14)[2],del(3)(p21)[2],del(3)(q13)[6],del(3)(q22)[3],
	der(3)ins(3;5)(p14;q13q31)[2],der(3)del(3)(p13)del(3)(q13q25)ins(3;5)(q13;q13q31)[2],
	-4[19],-5[2],+5[3],-6[17],+7[3],del(7)(p21)[3],del(7)(p13p14)[5], del(7)(p13p14)x2[10],del(7)(p13p15)[8],der(7)t(7;15)(q21;q13)[3],dup(7)(p13p14)[2],+8[12],der(8;14)(q10;q10)x2[24],-9[11],-9x2[9],-10[11],-10x2[10],del(10)(p10)[3], der(10)t(3;10)(q?;q24)del(10)(p11.2)[14],der(10)t(3;10)(q?;q24)del(10)(p11.2)x2[10],+11[9],+11x2[7],+11x3[2],der(11)t(11;17)(q23;q?)t(9;17)(q?12;?)[2],-12[2],+12[6],+12x2[4],
	del(12)(p12)[6],del(12)(q24.1)[5],del(12)(q24.1)x2[3],der(12)del(12)(p12)del(12)(q24)[4],
	der(12)t(12;13)(p12;q22)[10],der(12)t(12;16)(p11.2;?)[11],-13[16],-13x2[4],+14[3],+14x2[13],
	+14x3[3],-15[6],-15x2[18],-16[2],der(16)t(1;16)(q12;q12)dup(1)(q21q43)[24],
	dic(9;17)t(9;17)(p12;p13)[13],dic(9;17)t(9;17)(p12;p13)x2[11],-18[17],-18x2[4],-19[18],
	+20[9],+20x2[3],der(20)t(10;20)(q21;q13.3)[15],der(20)t(10;20)(q21;q13.3)x2[9],der(20)del(20)(p11)t(10;20)(q21;q13.3)[10],+21[10],+21x2[6],-21[2], -22[14][cp24]
**BT474**	65 ~ 106 < 4n>,X,-X[9],-Xx2[5],-Xx3[4],der(X)t(X;17)(q13;q11q12)del(X)(p21)
	[9],der(X)t(X;18;X;12)[2],del(X)(q22)[14],-1[6],-1x2[2],+1[3],del(1)(p36.1)[6], -2[7],+2[7],der(2)t(1;2;7;20)(?;q31;?;?)[18],+3[12],-3[3],del(3)(p11.2)[7],
	del(3)(p14)[2],del(3)(q11.2)[6],del(3)(q11.2)x2[8],del(3)(q21)[4],del(3)(q13)[2],
	-4[8],-4x2[9],+4[2],-5[9],-5x2[9],+6[11],+6x3[3],-6[3],
	del(6)(q13)[3],del(6)(q21)[3],der(6)t(6;7)(q25;q31)[7],der(6)t(6;7)(q25;q31)x2[16],+7[4],+7x2[6],+7x3[9],+7x4[3],der(7)t(7;20)(p13;?)[5], der(7)t(1;7)(?;q11.2)[9],
	del(7)(q11.2)[7],del(7)(q11.2)x2[3],del(7)(q11.2)x3[3],der(7)t(7;14)(p13;p11.2)[4],-8[10], -9[7],-9x2[4],-9x3[2],der(9)t(3;9)(q33;?)[3],+10[6],-10[5],
	der(10)t(10;16;19)(q25;?;?)[11],i(10)(q10)[4],+11[9],+11x2[2],-11[3],
	der(11)t(8;11)(q21.1;p15)[2],der(11)t(8;17)(q21.1;q11q12)t(11;17)(p15;q11q12)[8],der(11)t(8;17)(q21.1;q11q12)t(11;17)(p15;q11q12)x2[12],der(11)t(8;17)(q21.1;q11q12)t(11;17)(p15;q11q12)x3[3],der(11)t(11;17)(q?14;?)t(8;17)(?;q?11.2)[13], der(11)t(11;17)(q?14;q?11.2)[9],+12[8],
	+12x2[5],del(12)(p11.1)[2],der(12)t(5;12)(q23;q23)[17],der(12)t(5;12)(q23;q23)x2[2],der(12)del(12)(p12)del(12)(q24)[3],-13[7],+13[6],+13x2[3],+13x4[2],
	der(13)t(13;17)(q10;q11q12)t(13;17)(q10;q11q12)
	[8],der(13)t(13;17)(q10;q11q12)t(13;17)(q10;q11q12)x2[12],+14[11], +14x2[3],+14x3[2],der(14)t(14;1;14)(q31;?;?)[6],der(14)t(14;1;14)(q31;?;?)x2[5],
	der(14)t(14;1;14)(q31;?;?)x3[9],der(14)t(14;1;14)(q31;?;?)x4[3],
	add(14)(p11.2)[2],der(14;14)(q10;q10)[3],der(14;14)(q10;q10)x2[16],-15[6],-15x2[9], -15x3[6],+16[7],+16x2[6],+16x3[3],-16[2],der(16)t(X;16)(q22;q24)[10],
	+17[16], der(17)t(6;17)(?;p13)t(15;17)(q11.2;q25)[22],-18[10],-18x2[4],-18x3[2],-19[6],
	-19x2[5],+19[5],-20[6],-20x2[6],+20[3],+20x3[2],der(20)t(19;20)(?;q10)[4],
	der(20)t(19;20)(?;q10)x2[5],+21[2],-21x2[11],-21x3[3],-22[2],-22x2[5],-22x3[2],-22x4[12],
	der(22)t(16;22)(q12;p11.2)[5][cp23]
**SKBR3**	76 ~ 83 < 4n>,XXX,-X[19],der(X)t(X;17)(q21;q?21)[15], der(X)t(X;8;17)(q13;q?21;?)[6],+1[8],+1x3[5],add(1)(p36.3)[4],
	del(1)(p13)[11],del(1)(p13)x2[6],del(1)(p34)[4],del(1)(p22)[9],del(1)(p36.1)[2], der(1)t(1;4)(q12;q12)[6],-2[6],-2x2[8],-2x3[3],der(2)t(2;6)(p13;?)[5],-3[10],-3x2[6],-4[8],
	-4x2[8],-4x3[3],der(4;14)t(4;14)(p11;p11.1)[3],-5[8],
	-5x2[8],-5x3[2],der(5)ins(5;15)(p13;q12q22)[6],-6[4],-6x2[12],
	-6x3[2],der(6)t(6;14;17)(q21;?;q11q12)del(6)(p23)[8],+7x2[8],+7x3[10],
	del(7)(q22)[12],del(7)(q32)[3],dup(7)(p14p15)[2],-8[6],+8[8],
	der(8)t(8;21)(?;?)t(8;21)(p23;?)t(8;21)(q24;?)[11],der(8)t(8;21)(?;?)t(8;21)(p23;?)t(8;21)
	(q24;?)x2[8],der(8)dup(8)(?)t(8;8)(?;p23)t(8;17)(q24;?)t(11;17)(?;?)[4],
	der(8;14)t(8;14)(p11.1;p11.1)[15],-9[9],-9x2[7],-10[4],-10x2[13],-10x3[2],+11[2],-11[7],
	add(11)(p15)[4],add(11)(q25)[2],-12[6],-12x2[5],+12[3],der(12)t(11;12)(p?;p12)[4],
	der(12)t(5;12)(q23;q23)[10],der(12)t(5;12)(q23;q23)x2[4],-13[6],-13x2[8],
	-13x3[3],der(13;13)(q11.2;q11.2)[16],-14[6],-14x2[4],
	der(14;14)(q11.2;q11.2)[18],-15[10],-15x2[7], dic(15;21)(p11.1;p11.1)[3],
	+16[4],-16[7],-17[3],+17[9],der(17;17)t(17;17)(q25;?)dup(17)(q22q25)t(17;20)(?;?)[5],
	der(17;17)t(17;17)(q25;?)dup(17)(q22q25)t(17;20)(?;?)x2[7], der(17;17)t(17;17)(q25;?)dup(17)(q22q25)t(17;20)(?;?)x3[7],del(17)(p11.2)[7],
	der(17)t(8;17)(q12;?)dup(17)(?)[19],der(17)t(8;17)(?;q25)dup(17)
	(q22q25)[5],der(17)t(8;17)(?;q25)dup(17)(q22q25)x2[2],der(17)t(8;13;14;17;21)(?;q?;q?;q11q12;?)[8],der(17)t(3;8;13;17;20)(?;?;q12;?p;?)[12],der(17)t(3;8;13;17;20)(?;?;q12;?p;?)x2[2],-18[3],-18x2[11],-18x3[5],der(18)t(18;22)(p11.2;?)[12],-19[4],-19x2[7],-20[8],-20x2[4],
	-20x3[7],-21[6],-21x2[3],-22[9],-22x2[4],+22[2],der(22)t(19,22)(q?;q13)[5][cp19]

The number of metaphases analyzed is reported in brackets at the end of each karyotype. Additionally, the frequency of each rearrangement identified is described in brackets.

Less frequent alterations (mainly numerical) constituted cluster 3 (green bar), and very rare alterations (ranging from 0 in metaphases M_21 and M_26 to 5 in metaphases M_13 and M_22) constituted cluster 4 (purple bar).

### Cytogenetic profile and cluster analysis of T47D cells

In the T47D cells, 24 metaphases were examined. The modal number was near triploidy (3n+/-) (57 and 66 chromosomes). T47D cells had 52 different chromosomal alterations (27 numerical and 25 structural) (Figure 1). Polyploidy was observed in 4% of the analyzed cells, and numerical chromosomal alterations were present in all chromosomes. Structural aberrations (deletions, translocations, and duplications) were found in all chromosomes except 2, 4, 18, 19, 21 and 22.

**Figure 4:**
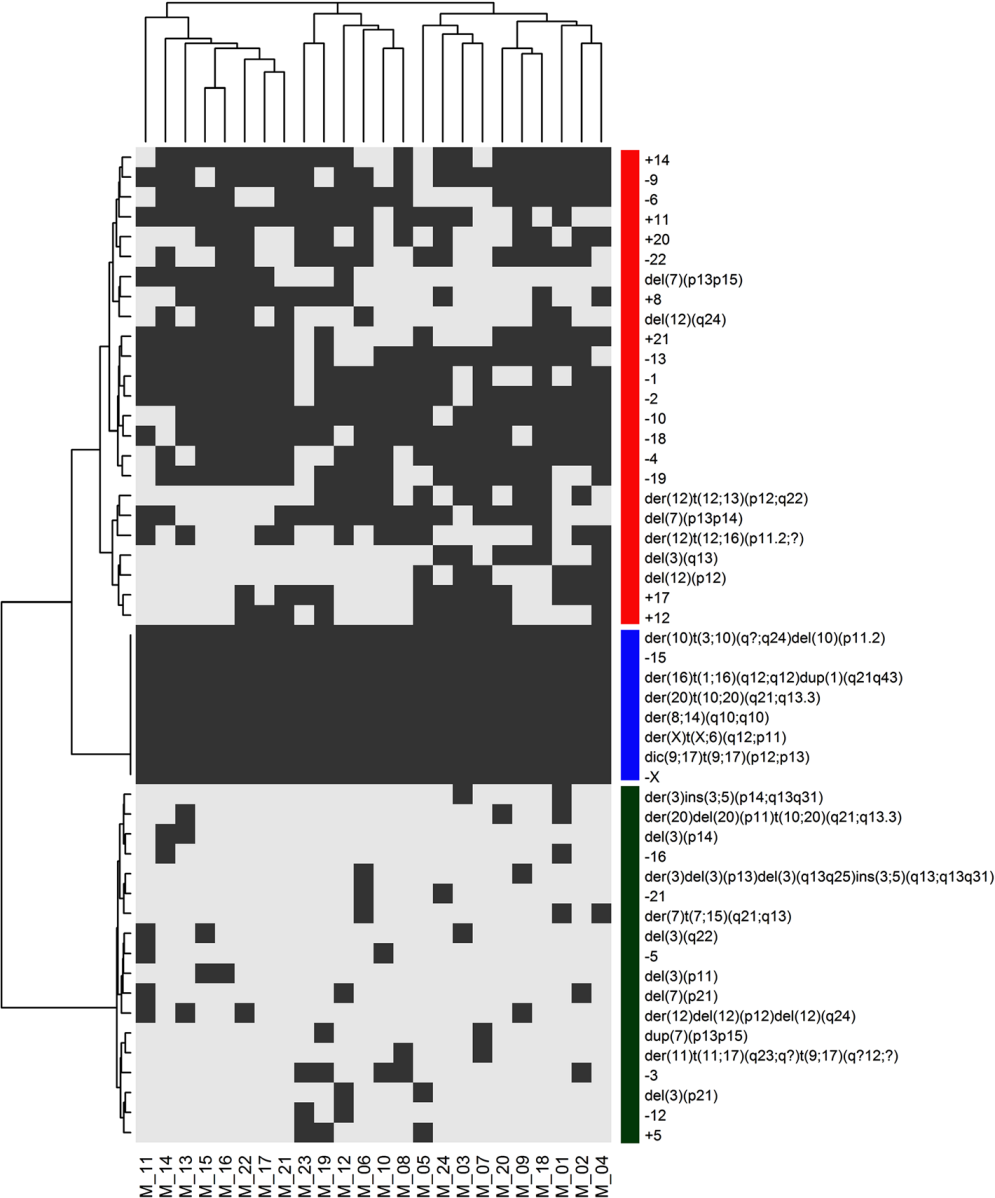
**Hierarchical cluster analysis of the presence or absence of chromosomal aberrations observed in 24 T47D metaphases.** Each column refers to a metaphase (M) and each row to a chromosomal abnormality. Grey indicates the presence of each abnormality, and white indicates their absence. The cluster number is indicated by vertical color bars. Cluster 1: red bar, cluster 2: blue bar and cluster 3: green bar.

As in the MCF7 cells, the types of chromosomal alterations were almost homogeneously distributed among the 24 metaphases of T47D cells, as demonstrated by hierarchical clustering (horizontal dendrogram, Figure 4). When the frequency of chromosomal alterations was analyzed, 3 clusters were identified (vertical dendrogram): the first and largest cluster (red bar) was formed by common numerical alterations with a prevalence of losses. The rare structural aberrations present in this cluster primarily involved chromosome 12. In the second cluster (the smallest, blue bar), der(X)t(X;6)(q12;p11), der(8;14)(q10;q10), der(10)t(3;10)(q?;q24)del(10)(p11.2), der(16)t(1;16)(q12;q12)dup(1)(q21q43), dic(9;17)t(9;17)(p12;p13) and der(20)t(10;20)(q21;q13.3) were present in all metaphases as the result of translocations, together with the loss of chromosomes 15 and X (Table 1 and Figure 3C and 3D). Cluster 3 (green bar) grouped rare abnormalities (ranging from zero in metaphases M_17 and M_21 to 4 in metaphases M_11 and M_10), most of which were structural (Figure 4).

### Cytogenetic profile and cluster analysis of BT474 cells

For BT474 cells, 23 metaphases were examined. These cells showed the highest frequency of numerical and complex structural aberrations of all cell lines analyzed. BT474 cells had a modal number near tetraploidy (4n+/-) (from 65 to 106 chromosomes) and showed 35 numerical and 36 structural aberrations (Figure 1). Polyploidy was not present.

**Figure 5:**
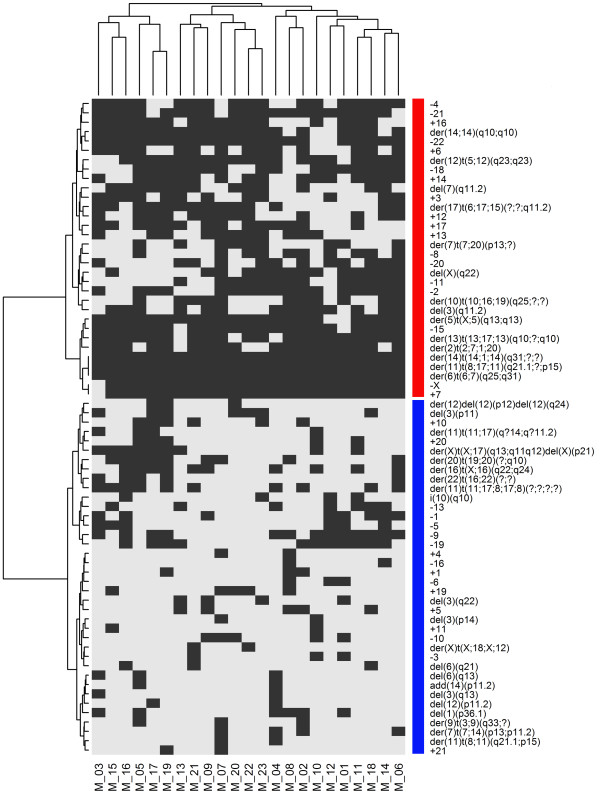
**Hierarchical cluster analysis of the presence or absence of chromosomal aberrations observed in 23 BT474 metaphases.** Each column refers to a metaphase (M) and each row to a chromosomal abnormality. Grey indicates the presence of each abnormality, and white indicates their absence. The cluster number is indicated by vertical color bars. Cluster 1: red bar and cluster 2: blue bar.

As in the other cell lines, cluster analysis demonstrated nearly homogeneous chromosome alterations in all metaphases (horizontal dendrogram, Figure 5). Isochromosomes, deletions and derivatives were frequent (Table 1 and Figure 3E and 3F). Numerical alterations were also observed in all chromosomes, with losses being more frequent than gains. Losses of chromosomes X, 15 and 22 were observed in 78%, 91% and 91% of metaphases, respectively, while gain of chromosome 7 was identified in 96% of cells.

The frequency of alterations within the cell line produced 2 clusters (vertical dendrogram): in cluster 1 (red bar), both numerical and structural alterations were present in almost all cells. Only three structural alterations were reproduced in all metaphases, namely der(6)t(6;7)(q25;q31), der(11)t(8;17;11)(q21.1;?;p15) and der(14;1;14)(q31;?;?) (Table 1 and Figure 3E and 3F). Cluster 2 (blue bar) included sporadic aberrations with a minimum of 3 such alterations observed in metaphase M_22 (Figure 5).

### Cytogenetic profile and cluster analysis of SKBR3 cells

In this cell line, 19 metaphases were examined. SKBR3 cells showed a hypertriploid to hypotetraploid (4n+/-) (76 to 83 chromosomes) karyotype. Polyploidy was observed in 19% of all cells. SKBR3 cells had 29 numerical and 33 structural aberrations (Figure 1). Numerical chromosomal alterations were observed in all chromosomes. Structural aberrations (translocations, deletions, and duplications) were found in all chromosomes except 3, 9, 10 and 16 (Table 1 and Figure 3G and 3H).

**Figure 6:**
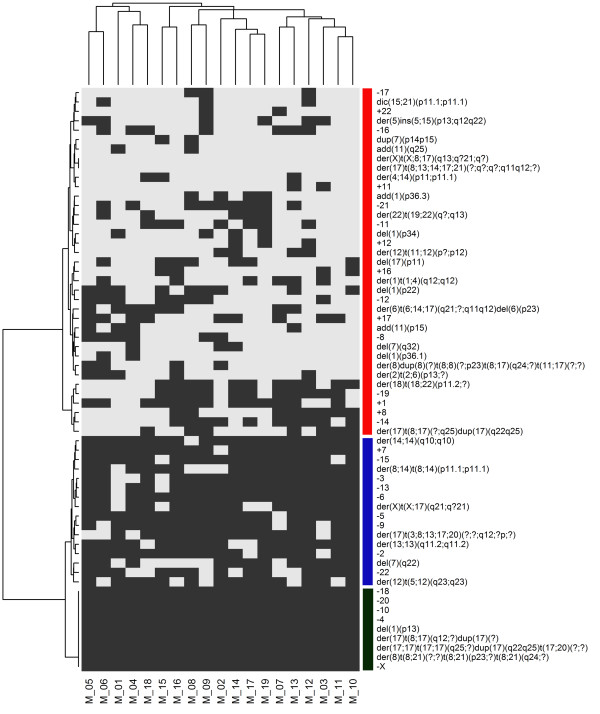
**Hierarchical cluster analysis of the presence or absence of chromosomal aberrations observed in 19 SKBR3 metaphases.** Each column refers to a metaphase (M) and each row to a chromosomal abnormality. Grey indicates the presence of each abnormality, and white indicates their absence. The cluster number is indicated by vertical color bars. Cluster 1: red bar, cluster 2: blue bar and cluster 3: green bar.

In comparison to other cell lines, hierarchical clustering showed similarities of chromosomal alterations among the 19 metaphases (horizontal dendrogram, Figure 6). Clustering by the frequency of chromosomal alterations defined 3 clusters (Figure 6). The largest cluster (cluster 1, red bar) was formed by sporadic aberrations, with structural aberrations being prevalent. Cluster 2 (blue bar) included frequent rearrangements, with more numerical than structural aberrations. The smallest group (cluster 3, green bar) contained chromosomal abnormalities that were present in all cells, both numerical, such as monosomies of chromosomes X, 4, 10, 18 and 20, and structural, such as those on chromosomes 8, 17 and 1.

### Comparison of the four cell lines

**Figure 7:**
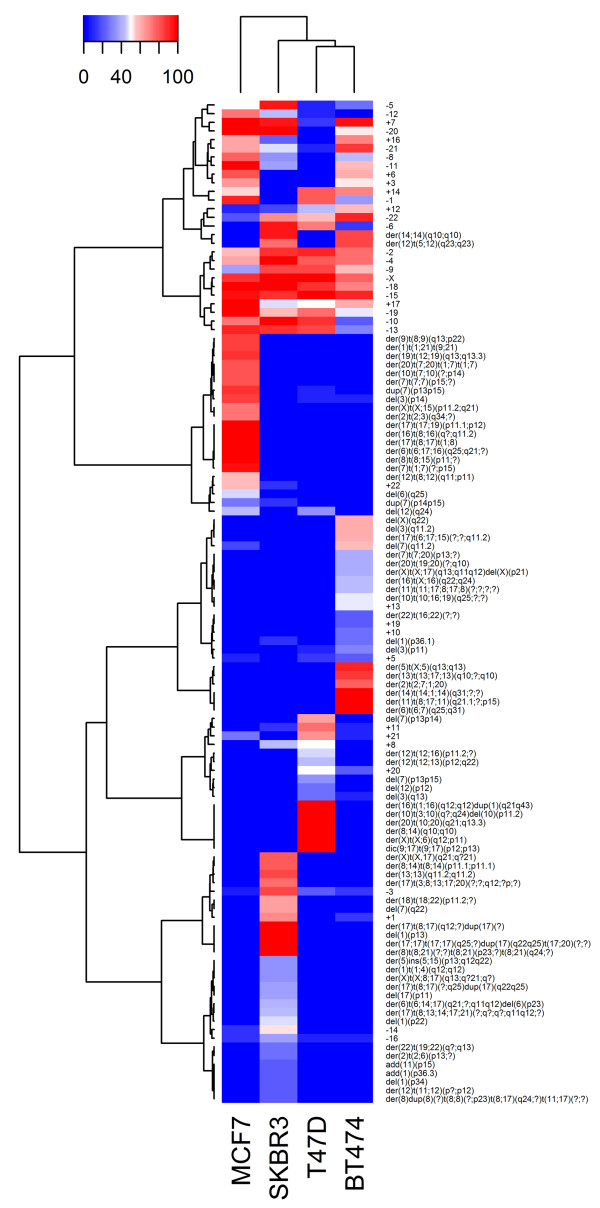
**Hierarchical cluster analysis of the percentage of chromosomal aberrations observed in four breast cancer cell lines.** Clustering stratifies cell lines into five groups. The first cluster was characterized by the presence of numerical chromosomal abnormalities (aneuploidies) that were common to the four cell lines (ER+, ER-, HER2+, HER2-). The other clusters comprised cell type-specific chromosomal abnormalities. The gradient color indicates percentage of chromosomal abnormalities present in each cell line.

**Figure 8:**
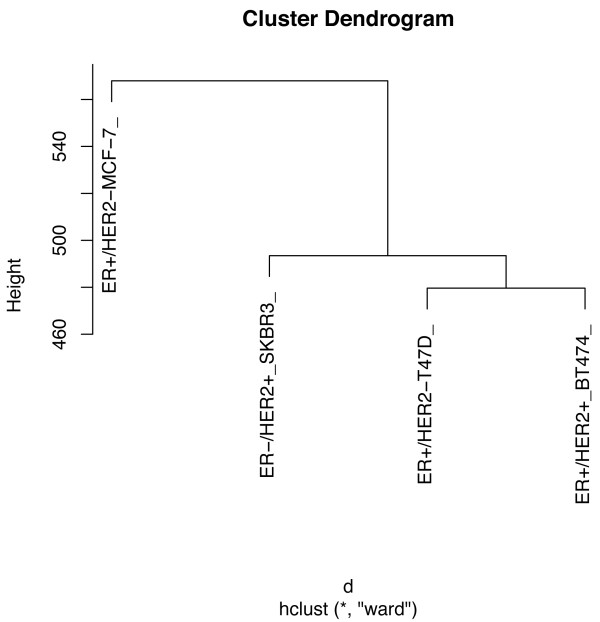
**Cluster dendrogram derived from cytogenetic analysis of the four breast cancer cell lines.** These analyses confirmed the greater similarities between T47D and BT474 cell lines and between these two cell lines and the SKBR3. MCF7 cells demonstrate a chromosomal pattern that was markedly different from those of previous cells.

Using hierarchical clustering, we identified five major clusters (Figure 7). One cluster was characterized mainly by numerical chromosome abnormalities (18 losses and 7 gains) that were common to the four cell lines. Only two structural alterations, namely der(14;14)(q10;q10) and der(12)t(5;12)(q23;q23), were common to HER2+ cells. The other clusters, however, encompassed cell type-specific abnormalities that were primarily structural (Figure 7). This analysis revealed greater similarity between T47D and BT474 cells and some similarity between these two cell lines and the SKBR3 cell line. MCF7 cells demonstrated a chromosome pattern that was markedly different from those of the other lines (Figure 8).

## Discussion

The MCF7 (ER+/HER2-), T47D (ER+/HER2-), BT474 (ER+/HER2+) and SKBR3 (ER-/HER2+) cell lines are widely used in breast cancer research as paradigms of the luminal and HER2 immunophenotypes [9,10]. Although classical cytogenetic analysis is time consuming and lacks the resolution of molecular techniques, it is the best tool for obtaining an overall picture of the types and frequency of chromosome changes. The results obtained using G-Banding and M-FISH analyses of a large number of metaphases allowed us to acquire a thorough insight of the type and frequency of chromosome alterations in the MCF7, T47D, BT474 and SKBR3 cell lines and to detect previously unreported chromosome alterations (Table 2).

**Table 2:** Comparison of selected chromosomal aberrations detected in MCF7, T47D, BT474 and SKBR3 cell lines in previous studies and in our G-banding and M-FISH results

**Cell line**	**ATCC**	**National Center for Biotechnology Formation NCBI [**[18]	**Gasparini, et al. 2010 [**[15]**]**	**Davidson, et al. 2000 [**[14]**]**	**G-banding and M-FISH present study**
MCF7	NR	NR	dup(X)(?;qter)	der(1)t(X;1)	der(X)dup(X)(q21qter)
	NR	NR	NR	NR	der(6)t(6;17;16)(q25;q21;?)
	NR	der(17)t(17;20)(q25;?)t(1;20)t(1;3or7)	NR	der(?)t(11;1;17;19;17)	der(17)t(17;19)(p11.1;p12)
	NR	NR	NR	der(?)t(17;1;19;17;20)	der(17)t(8;17)t(1;8)
T47D	der(8)t(8;14)	der(8)t(8;14)(p21;q21)	_	der(8)t(8;14)	der(8;14)(q10;q10)
	der(9)t(9;17)	der(9)t(9;17)(p12;q?11)	_	NR	dic(9;17)t(9;17)(p12;p13)
	der(10)t(10;20)	der(20)t(10;20)(q21;q13)	_	NR	der(20)t(10;20)(q21;q13.3)
BT474	der(6)t(6;7)(q21;q21)	_	der(6)t(6;7)(q25;?)	_	der(6)t(6;7)(q25;q31)
	NR	_	der(11)t(8;11;??)(?;p15;?)	_	der(11)t(8;17;11)(q21.1;?;p15)
	NR	_	NR	_	der(11)t(11;17)(q?14;q?11.2)
	i(13q)	_	der(13;13)(q10;q10)	_	der(13)t(13;17;13)(q10;?;q10)
	der(14)t(14;?)(q32,?)	_	der(14)t(1;14;X)(?;q31;?)	_	der(14)t(14;1;14)(q31;?;?)
SKBR3	NR	_	NR	der(8)t(8;21)	der(8)t(8;21)(?;?)t(8;21)(p23;?)t(8;21)(q24;?)
	NR	_	NR	NR	der(8)dup(8)(?)t(8;8)(?;p23)t(8;17)(q24;?)t(11;17)(?;?)
	NR	_	NR	der(?)t(8;14)	der(8;14)t(8;14)(p11.1;p11.1)
	NR	_	NR	NR	der(17)t(8;17)(q12;?)dup(17)(?)
	NR	_	NR	der(?)t(20;19;8;17)	der(17;17)t(17;17)(q25;?)dup(17)(q22q25)t(17;20)(?;?)
	NR	_	NR	der(8?)t(13;3;8;3;8;13)	der(17)t(8;13;14;17;21)(?;q?;q?;q11q12;?)
	NR	_	NR	der(?)t(20;3;8;17;19;8;3;13)	der(17)t(3;8;13;17;20)(?;?;q12;?p;?)
	NR	_	NR	NR	der(17)t(8;17)(?;q25)dup(17)(q22q25)
	NR	_	NR	der(?)t(19;22)	der(22)t(19,22)(q?;q13)

*Abbreviations*: NR, not reported. Dashes indicate that no information was available.

Cluster analysis excluded the presence of cell clones within each cell line because the same abnormalities were homogenously observed in all metaphases. Conversely, within the same cell line, the frequency of each chromosome alteration was variable and defined different clusters. Finally, a comparison of these four cell lines using cluster analysis showed that they shared up to 5 numerical aberrations in more than 50% of the metaphases (-2, -4, -15, -18, -X) and that the chromosomal structural alterations were cell-type specific, with the exception of two derivative chromosomes that were shared by the BT474 and SKBR3 HER2+ cell lines.

The HER2+ cell lines BT474 and SKBR3 showed the highest frequency of numerical and structural aberrations in comparison with the HER2- cell lines MCF7 and T47D. Polyploidy, which was more frequent in HER2+ than in HER2- cells, has been correlated with short survival, drug resistance and metastasis [19]. In addition, complex chromosome alterations affecting chromosomes 8, 11, and 17 were frequently observed in HER2+ cells. These chromosomes contain genes that are commonly involved in the invasion, metastasis and pathogenesis of breast cancer, including *c-MYC* on 8q24; *HRAS, CD151, CTSD* on 11p15; *CCND1* on 11q13 [20-24]; and *TOP2A* on 17q21. Moreover, in HER2+ cells and carcinomas, rearrangements of chromosome 17 are more frequent than is polysomy. Pathologists must consider this observation for when diagnosing the HER2 amplification in interphase nuclei of breast carcinomas, which uses a ratio between HER2 copies and chromosome 17 centromere signals [25,26].

Among ER + cells, MCF7 cells are cytogenetically different than both T47D (ER+/HER2-) and BT474 (ER+/HER2+) cells and are characterized by a specific subset of complex structural alterations, which are listed in the cluster analysis comparison of the four cell lines (Figure 7). In particular, chromosome 7 was frequently structurally and numerically affected, and polysomy of chromosome 7 was observed in all metaphases. This finding has been closely associated with lymph node metastasis and prognosis in breast cancer patients [27]. One may speculate that the differences observed in the pattern of chromosomal aberrations between the MCF7 and T47D cell lines could partly explain the differences in the profile of protein expression that was recently identified in these cells [28]. Proteomic studies have revealed that a high number (at least 164) of proteins (including proteins involved in the regulation of breast cancer cell growth) are differentially expressed by T47D and MCF7 cells [28]. For example, of the proteins that are principally involved in cell proliferation and apoptosis and are upregulated in MCF7 cells, the Chromobox protein homolog 3 and the Cytochrome c-releasing factor 21 are encoded by genes mapping to chromosome 7, which is typically polysomic in MCF7 cells, as reported above. The differences in the karyotype should be considered when designing related experimental studies, such as those that analyze the effect of gene transfection. It is possible that complex chromosome alterations may alter the results. MCF7 cells, which differ greatly from the BT474 and SKBR3 (HER2+) cells, are frequently used to study the effect of HER2 transfection [29-31]; however, they may not represent the best substrate*.* Conversely, T47D cells (ER+/HER2-) and BT474 cells share similarities in the chromosome profile, and both have some chromosomal similarities with SKBR3 cells. For example, T47D and BT474 cells share numerical alterations, such as losses of chromosome 6 and gains of chromosomes 11 and 20, but they have no structural abnormalities in common.

One may hypothesize that the earliest genetic event may be aneuploidy, followed by structural alterations [32,33]. Aneuploidy is one of the most common properties of cancer [34]. In addition, numerical abnormalities have been observed more frequently in primary cancers, while structural alterations and amplifications were more commonly observed in metastatic breast cancer [33]. These structural alterations may lead to the deregulated expression of genes, such as a loss of tumor suppressor genes, the activation of oncogenes and the formation of fusion proteins with enhanced or aberrant transcriptional activity. For instance, some of the genes upregulated in HER2+ cell lines [35] reside on chromosomes 5, 6, 10, 19, and 20, which were reported to be polysomic in BT474 cells in the present study (Additional file 1: Table S1).

## Conclusions

In conclusion, by using both conventional and molecular karyotyping, our work provides a comprehensive and specific characterization of complex chromosomal aberrations for MCF7, T47D, BT474 and SKBR3 cell lines, thus providing important information for experimental studies. These cell lines serve as models for investigating the molecular biology of breast cancer; therefore, it may be essential to consider the potential influence of these chromosomal alterations when interpreting biological data.

## Methods

### Cell lines

The human breast cancer cell lines MCF7 (ER+/HER2*-*), T47D (ER+/HER2*-*), BT474 (ER+/HER2*+*) and SKBR3 (ER-/HER2*+*) were obtained from the American Type Culture Collection (ATCC, Manassas, VA, USA) in March 2010. Short tandem repeat (STR) analysis is routinely performed by ATCC during both accessioning and culture replenishment to avoid distributing misidentified cell lines to the scientific community. When received by our lab, these cell lines were expanded, and 3 vials were immediately frozen. Cells obtained from these stocks were used for the experiments. The cell lines were further authenticated based on the expression of epithelial markers (keratins 8 and 18) and the presence of specific receptors (ERα, PGR, HER2, AR and EGFR) using quantitative PCR (qPCR) and immunohistochemical analysis. The expression status of ERα and HER2 was further confirmed by western blot.

MCF7, T47D, and SKBR3 cells were cultured in RPMI 1640 medium (Sigma, St. Louis, MO, USA), while BT474 cells were cultured in DMEM medium (Sigma). Culture media were supplemented with 10% fetal bovine serum (FBS) (Sigma), antibiotic-antimycotic solution (1X) (Sigma) and L-glutamine (2 mM) (Invitrogen GmbH, Karlsruhe, Germany). The cultures were maintained in an incubator at 37°C and 5% CO2 and were determined to be free of contamination with mycoplasma by PCR assay. Cell line characteristics and culture conditions are further described in supplemental information (Additional file 2: Table S2).

### Metaphase spreads and G-Banding

Metaphases were obtained using standardized harvesting protocols for conventional and molecular cytogenetic analysis (M-FISH). Briefly, colcemid solution (0.03 μg/ml) (Sigma) was added to cultures 2.5 hours (h) before cell harvesting; cells were then treated with hypotonic solution, fixed three times with Carnoy’s fixative (3:1 methanol to acetic acid) and spread on glass.

Glass slides were baked at 70°C for 24 h, incubated in HCl and placed in 2xSSC buffer before treatment with Wright’s stain. Image acquisition and subsequent karyotyping of metaphases were performed using a Nikon microscope with the cytogenetic software CytoVision System (Applied Imaging, Santa Clara, CA, USA). Chromosome aberrations were described according to the International System for Human Cytogenetic Nomenclature (ISCN) 2013 [36].

### Multi-color FISH (M-FISH)

M-FISH was performed with the aim of identifying complex chromosomal rearrangements. The probe cocktail containing 24 differentially labeled chromosome-specific painting probes (24xCyte kit MetaSystems, Altlussheim, Germany) was denatured and hybridized to denatured tumor metaphase chromosomes according to the manufacturer’s protocol for the Human Multicolor FISH kit (MetaSystems). Briefly, the slides were incubated at 70°C in saline solution (2xSSC), denatured in NaOH, dehydrated in ethanol series, air-dried, covered with 10 μl of probe cocktail (denatured) and hybridized for two days at 37°C. The slides were then washed with post-hybridization buffers, dehydrated in ethanol series and counter-stained with 10 μl of DAPI/antifade. The signal detection and analysis of subsequent metaphases used the Metafer system and Metasytems’ ISIS software (software for spectral karyotypes).

### Hierarchical clustering

The first cluster analysis was performed to assess the chromosomal heterogeneity of each cell line by considering the type and frequency of chromosomal alterations within metaphases. Each alteration was computed as present or absent within the karyotype of different metaphases. In the second cluster analysis, the frequency (%) of each chromosomal alteration was compared among the four cell lines. Hierarchical clustering was performed using package gplots from the Bioconductor project (http://www.bioconductor.org) for the R statistical language. A Euclidean distance was used to calculate the matrix of distances, and clusters were built using Ward’s method.

## Abbreviations

CGH: Comparative genomic hybridization; aCGH: Array comparative genomic hybridization; MLPA: Multiplex ligation dependent probe amplification; ER: Estrogen receptor; M-FISH: Multicolor fluorescence *in situ* hybridization; AC: Adenocarcinoma; IDC: Invasive ductal carcinoma; PE: Pleural effusion; FBS: Fetal bovine serum; DMEM: Dulbecco’s modified Eagle’s medium.

## Competing interests

The authors declare that they have no competing interests.

## Authors’ contributions

All authors made substantial contributions to conception and design, analysis and interpretation of data, and critical review of the manuscript. AS, MRL, LV and CM conceived the study, coordinated the data acquisition and analysis, and co-wrote the manuscript. MRL, LV, NR, PG and CB coordinated and performed the experiments. MRL, NR and CP performed the biostatistical and hierarchical cluster analysis. SR, GB and BP provided assistance with manuscript preparation. All authors read and approved the final manuscript.

## Supplementary Material

Additional file 1: Table S1 Upregulated and downregulated genes in HER2+ breast cancer cell lines reported by Wilson, et al. (2002) [35] and located in the chromosomal region observed to be altered in this study and significantly associated with this group. Click here for file

Additional file 2: Table S2 Characteristics of breast cancer cell lines. Data obtained from ATCC. Click here for file
