# Supplementary material for: Differences and homologies of chromosomal alterations within and between breast cancer cell lines: a clustering analysis
Source: Mol Cytogenet. 2014 Jan 23;7:8. doi: 10.1186/1755-8166-7-8 (PMC3914704; doi:10.1186/1755-8166-7-8)
Supplement: Additional file 1: Table S1 — Upregulated and downregulated genes in HER2+ breast cancer cell lines reported by Wilson, et al. (2002) [35] and located in the chromosomal region observed to be altered in this study and significantly associated with this group. [file 1755-8166-7-8-S1.doc]

**Table S1**. Up-Regulated and Down-regulated Genes in HER2+ breast cancer cell lines reported by Wilson, et al (2002) and located in the chromosomal region observed altered in this study and significantly associated with this group.

| **Gene** | **Up regulated** | **Down Regulated** | **Chromosome Localization** | **Type of Rearrangement present study** |
| --- | --- | --- | --- | --- |
| Endothelial differentiation sphingolipid G-protein-coupled receptor 1 |  | X | 1pter-qter | del(1)(p13) |
| High-mobility group protein 17 |  | X | 1p36 | del(1)(p36.1) |
| Complement 1q |  | X | 1p36 | del(1)(p36.1) |
| 1-Catenin (5q31) | X |  | 5q31 | +5 |
| Dihydropyrimidinase-like 3 | X |  | 5q32 | +5 |
| Heat shock protein 70 (HSP70) | X |  | 6p21 | +6 |
| Tumor protein D52 | X |  | 8q21 | der(8)dup(8)(?)t(8;8)(?;p23)t(8;17)(q24;?)t(11;17)(?;?) |
| Transforming acidic coiled coil containing protein 1 (TACC1) |  | X | 8p11 | der(8;14)t(8;14)(p11.1;p11.1) |
| Discs (Drosophila), large homolog 5 | X |  | 10q23 | +10 |
| Ribosomal protein S24 | X |  | 10q22-23 | +10 |
| Mammaglobin 1 | X |  | 11q13 | der(11)t(11;17)(q?14;q?11.2) |
| Tumor protein D52 | X |  | 8q21 | der(11)t(8;11)(q21.1;p15) |
| Hepatocyte nuclear factor 3a (HNF3a) | X |  | 14q12-13 | der(14;14)(q10;q10) |
| Proteasome subunit 􏰃6 | X |  | 14q13 | der(14;14)(q10;q10) |
| Oxidase (cytochrome c) assembly-like 1 | X |  | 14q11.2 | der(14;14)(q10;q10) |
| Poly(A)-binding protein 2 | X |  | 14q11.2 | der(14;14)(q10;q10) |
| Glycogen phosphorylase isotype BB |  | X | 20p11.2 | der(17;17)t(17;17)(q25;?)dup(17)(q22q25)t(17;20)(?;?) |
| LIM and SH3 protein 1 | X |  | 17q11-21.3 | der(17)t(8;17)(q12;?)dup(17)(?) |
| Ribophorin II | X |  | 20q12-13 | der(17)t(3;8;13;17;20)(?;?;q12;?p;?) |
| Ferritin, light polypeptide | X |  | 19q13 | +19 |
| Protein tyrosine phosphatase, non-receptor type substrate 1 | X |  | 20p13 | +20 |
