# Supplementary material for: Differences and homologies of chromosomal alterations within and between breast cancer cell lines: a clustering analysis
Source: Mol Cytogenet. 2014 Jan 23;7:8. doi: 10.1186/1755-8166-7-8 (PMC3914704; doi:10.1186/1755-8166-7-8)
Supplement: Additional file 2: Table S2 — Characteristics of breast cancer cell lines. Data obtained from ATCC. [file 1755-8166-7-8-S2.doc]

**Table S2.** Characteristics of Breast Cancer Cell Lines. Data obtained from ATCC.

| **Cell line** | **ATCC Code** | **P** | **Presence of Receptors** | ***HER2* gene status** | **Tissue source** | **Type of Tumor** | **Patient Age** | **Culture Conditions** |
| --- | --- | --- | --- | --- | --- | --- | --- | --- |
| MCF7 | HTB-22 | P146 | ER+ / PR+ | Not amplified | PE | AC | 69 | RPMI 1640 + 10% FBS + 2 mM L-glutamine + antimycotic-antibiotic solution (1X) |
| T47D | HTB-133 | P88 | ER+ / PR+ | Not amplified | PE | IDC | 54 |
| BT474 | HTB-20 | P86 | ER+ / PR+ | Amplified | DIC | IDC | 60 | DMEM + 10% FBS + 2 mM glutamine + antimycotic-antibiotic solution (1X) |
| SKBR3 | HTB-30 | P29 | ER- / PR- | Amplified | PE | AC | 43 | RPMI 1640 + 10% FBS + 2 mM L-glutamine + antimycotic-antibiotic solution (1X) |
| AC, adenocarcinoma; IDC, invasive ductal carcinoma; PE, pleural effusion; P, pass number. Culture conditions: FBS, Fetal bovine serum; DMEM, Dulbecco’s Modified Eagle Medium. Cell lines are maintained at 37ºC and 5% CO2 in the indicated medium. | | | | | | | | |
